# Appointments in the Medical College of Ohio

**Published:** 1833

**Authors:** 


### VIII. Appointments in the Medical College of Ohio.

Dr. Alban G. Smith, of Louisville, Kentucky, to the Professorship of Surgery, *vice* Doctor James M. Staughton, deceased.

Dr. Samuel D. Gross, of Easton, Penn. Demonstrator of Anatomy.

